# Dimensional Changes After Different Alveolar Ridge Preservation Techniques for Posterior Region: A Randomised Controlled Clinical Trial

**DOI:** 10.1111/jcpe.70004

**Published:** 2025-08-05

**Authors:** Leticia Sandoli Arroteia, Matheus Paschoaletto Lopes, Marcela Tarosso Réa, Thalita R. Vieira e Oliveira, Matheus L. Oliveira, Marcelo de Faveri, Mauro Pedrine Santamaria, Lucas Araujo Queiroz, Marcio Zaffalon Casati, Renato Corrêa Viana Casarin

**Affiliations:** ^1^ Periodontics Division, Department of Prosthodontics and Periodontics, Piracicaba Dental School University of Campinas Piracicaba SP Brazil; ^2^ Oral Radiology Division, Department of Oral Diagnosis, Piracicaba Dental School University of Campinas Piracicaba SP Brazil; ^3^ Department of Oral Surgery, School of Dental Medicine University at Buffalo Buffalo NY USA; ^4^ Periodontics Division, Department of Periodontics Guarulhos University Guarulhos SP Brazil; ^5^ Division of Periodontology, Center of Oral Health Research University of Kentucky Lexington KY USA; ^6^ Private Clinic São Luiz PI Brazil; ^7^ Department of Periodontics and Endodontics, School of Dental Medicine University at Buffalo Buffalo NY USA

**Keywords:** bone graft, cone‐beam computed tomography, dental implants, membranes, surgery

## Abstract

**Aim:**

Alveolar ridge preservation (ARP) after tooth extraction aims to maintain ridge dimensions for future implant placement. However, limited evidence is available for posterior sites. We evaluated the dimensional changes in posterior extraction sockets using four approaches: modified free gingival graft (MFGG); MFGG with xenograft (MFGG + XG); guided bone regeneration with titanium membrane and xenograft (TM + XG); and unassisted socket healing (USH).

**Materials and Methods:**

Patients requiring posterior tooth extraction were randomly assigned to one of four groups (*n* = 22/group). Cone‐beam computed tomography (CBCT) was performed immediately after operation and at 6 months to assess vertical and horizontal alveolar ridge changes and implant‐related outcomes.

**Results:**

Significant dimensional changes occurred in all groups (*p* < 0.05). The TM + XG and MFGG + XG groups showed better preservation of vertical ridge dimensions than the USH and MFGG groups (*p* < 0.05). Horizontal reductions were significant in all groups (*p* < 0.05). USH presented the highest sinus floor elevation rate (67% of maxillary cases) and the most frequent use of short implants (< 8.5 mm; 71%). Postoperative pain was significantly greater in the MFGG groups compared to the USH group (*p* < 0.05).

**Conclusion:**

Bone grafting with MFGG or a titanium membrane enhanced ridge preservation and reduced the need for additional surgery compared to USH.

**Trial Registration:** ClinicalTrials.gov identifier: NCT06081296

## 1 Introduction

Tooth loss affects up to 44.7% of adults (Flemming and Scharf 2016), and dental implants represent a highly successful therapeutic option (Duong et al. 2022). Implant success depends on adequate bone quantity and quality, which are crucial for long‐term outcomes. After tooth extraction, increased osteoclastic activity disrupts periodontal ligament fibres, leading to substantial buccal bone resorption (Bartold and Ivanovski 2022). In the anterior region, bone loss ranges from 1.44 to 2.73 mm (Couso‐Queiruga et al. 2021). While only a few studies have focused on the posterior region, remodelling is expected to result in 2.40–3.13 mm bone loss within 6 months (Festa et al. 2013). These changes, combined with the proximity of critical anatomical structures such as the maxillary sinus and inferior alveolar nerve canal, complicate posterior implant placement. Additionally, posterior ridge resorption affects soft‐tissue quality, leading to higher rates of peri‐implantitis and reduced rehabilitation success (Manor et al. 2009; Monje et al. 2023).

Modulating remodelling during healing is critical for optimising implant conditions. Alveolar ridge preservation (ARP) prevents excessive collapse of bone walls, maintaining ridge contours for implant placement and minimising the need for additional surgeries (Al Qabbani et al. 2018; Avila‐Ortiz et al. 2020; Couso‐Queiruga et al. 2023). Systematic reviews have confirmed that ARP effectively mitigates post‐extraction ridge alterations (Barootchi et al. 2023; Clark et al. 2018).

Among the ARP strategies, sealing sockets with membranes or soft‐tissue grafts stabilises the blood clot and compensates for soft‐tissue deficiencies, often without full‐thickness flaps (Karaca et al. 2015; López‐Pacheco et al. 2021; Canullo et al. 2022). Partially de‐epithelialised gingival grafts show outcomes comparable to collagen membranes for socket closure with bone grafting (Papace et al. 2021; Segnini et al. 2021) and contribute to improved peri‐implant health (Papace et al. 2021; Beretta et al. 2021).

Another ARP approach uses exposed occlusive membranes, which limit alveolar resorption by promoting keratinised tissue formation during healing (Anwandter et al. 2016; Avila‐Ortiz et al. 2020; Mardas et al. 2023). Recently, anodised non‐resorbable titanium membranes were introduced to preserve the regenerative space and maintain alveolar integrity in anterior regions (Maeda et al. 2020, 2021); however, evidence regarding their application in posterior sites remains limited.

Most ARP research has focused on the anterior region because of aesthetic concerns, underscoring the need for randomised clinical trials (RCTs) evaluating preservation techniques in posterior sites. This study aimed to address this gap by conducting an RCT assessing minimally traumatic extractions combined with ARP techniques in the posterior region.

## 2 Materials and Methods

### 2.1 Study Design

This single‐centre, randomized, controlled, superiority, single‐blind clinical trial compared tomographic dimensional changes following three ARP techniques: modified free gingival graft (MFGG); MFGG combined with xenograft (MFGG + XG); and titanium mesh with xenograft (TM + XG), against unassisted socket healing (USH) as the control. The study was approved by the Institutional Review Board (IRB) (CAAE: 59208422.8.0000.5418) and registered on ClinicalTrials.gov (ID: 59208422.8.0000.5418). This manuscript adhered to the CONSORT 2010 guidelines and the Declaration of Helsinki (1975; revised in 2013).

### 2.2 Study Population and Sample Size Calculation

Eighty‐eight patients from the Piracicaba Dental School (São Paulo, Brazil) requiring tooth extractions for non‐periodontal reasons in the premolar/molar regions of the maxilla or mandible were treated between September 2022 and January 2024. Inclusion criteria were (i) age over 18 years; (ii) at least two‐thirds of bone support for the extracted tooth; (iii) absence of significant periapical lesions; and (iv) signed informed consent. Exclusion criteria included (i) uncontrolled systemic diseases; (ii) periodontal disease at the time of surgery; (iii) less than 10% of bleeding on probing (BOP); (iv) less than 20% of plaque index (PI); (v) pregnancy or lactation; (vi) current or previous smoking; (vii) ongoing orthodontic treatment; and (viii) medications affecting bone healing (e.g., bisphosphonates). Exit criteria were (i) failure to attend the postoperative CT scan, and (ii) illness or use of medication interfering with healing. The sample size was calculated based on ridge height dimensions as the primary outcome. To achieve 80% power at a significance level of 0.05, with a standard deviation of 0.8 mm and difference among groups of 1.0 mm (Karaca et al. 2015), 17 patients per group (a total of 68) were required. To account for potential dropouts, 22 patients per group were recruited.

### 2.3 Preoperative Protocol

All patients received oral hygiene instructions and underwent scaling. Periodontal parameters were assessed by a calibrated examiner (L.S.A.; intra‐class correlation coefficient of 85% for probing depth [PD]) and included PI (Ainamo and Bay 1975), BOP (Mühlemann and Son 1971) and PD. Periapical radiographs were also obtained to confirm the extraction indication.

### 2.4 Randomization and Groups

**FIGURE 1:**
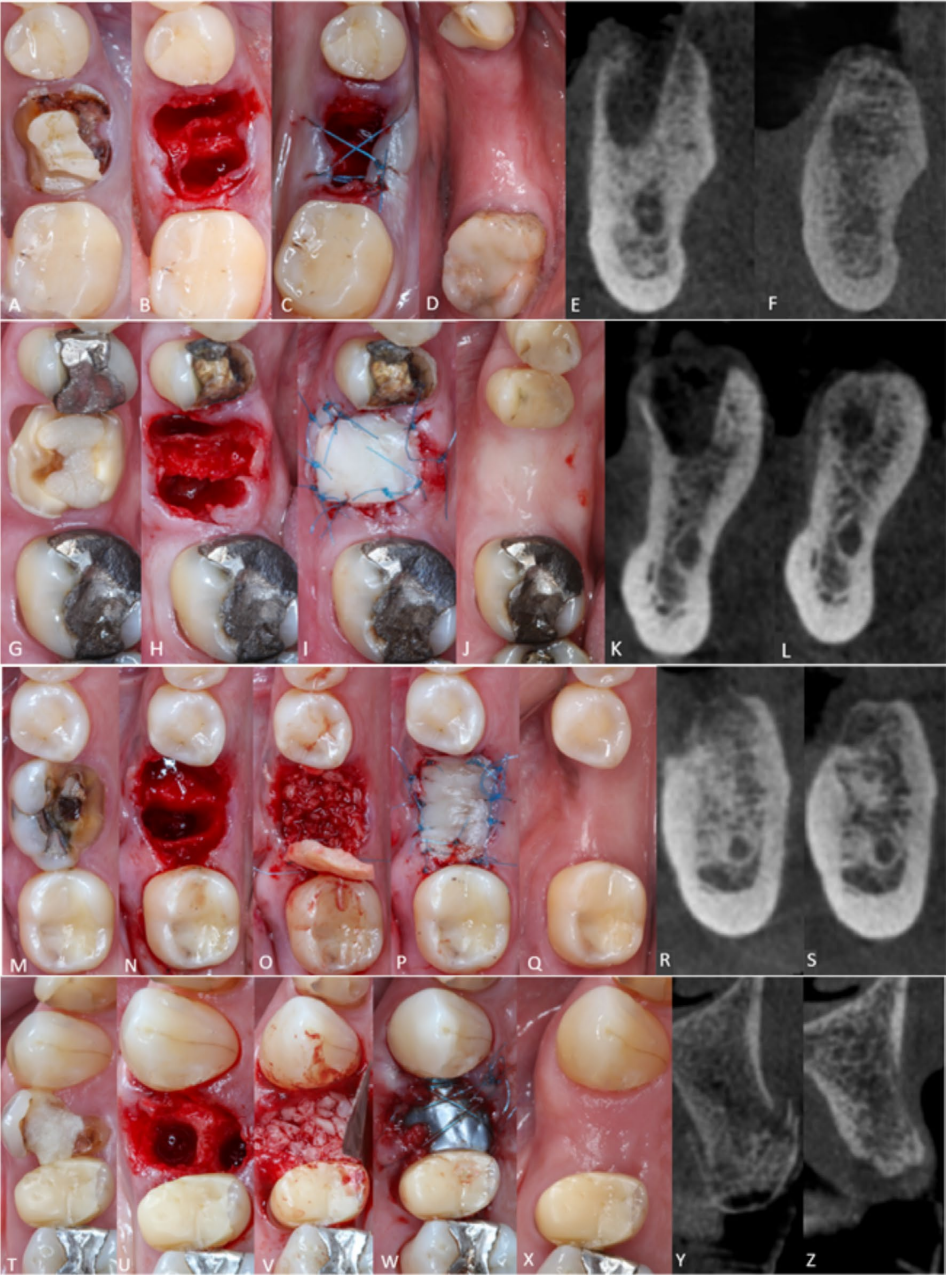
Clinical and tomographic images. *USH group*: (A) Initial view of the tooth to be extracted. (B) Alveolus immediately after minimally traumatic extraction. (C) Immediate aspect after suturing. (D) Healed socket after 6 months. (E) CBCT cross‐sections immediately after extraction and (F) after 6 months (G). *MFGG group*: Initial (H) and post‐extraction (I) MFGG sutured sealing the socket entrance. (J) Healed socket after 6 months. (K) CBCT cross‐sections immediately after extraction (L) and after 6 months; *MFGG + XG group*. Initial (M) and after extraction (N) view. (O) Alveolus filled with bovine bone graft and socket sealing with MFGG (P). (Q) Healed socket after 6 months. CBCT cross‐sections immediately after extraction (R) and after 6 months (S). *TM + XG group*: Initial view (T) and immediately after (U) minimally traumatic extraction. Bovine bone grafting (V) and sutures stabilising the titanium membrane (W); (X) Healed socket after 6 months; CBCT cross‐sections immediately after extraction (Y) and after 6 months (Z).

Randomization was performed following Sealedenvelope.com, and the treatment allocation code was placed in an opaque envelope, supervised by an independent operator (L.S.‐A.). Tooth extractions were carried out using a minimally traumatic technique by a single operator (R.C.V.C.). At the end of the extraction, sites were allocated to one of the study groups as follows:

– *Unassisted socket healing (USH; n = 22) group*: Atraumatic extraction was carried out using periotomes and delicate instruments. The socket was allowed to fill with a natural blood clot and was sutured with 5–0 nylon (Soft Blue NSB Techsuture—USP 5–0/Needle N 13 mm, 3/8 circle) (Figure 1A–F).
– *Modified free gingival graft (MFGG; n = 22) group*: Atraumatic extraction was performed, and the socket was allowed to fill with a blood clot, which was subsequently sealed using an MFGG without flap elevation. The FGG was harvested from the palate with standardised dimensions of 10 × 6 mm and, to optimise vascularisation and integration, the graft underwent a specific modification. First, 2 mm of the epithelium from both the buccal and lingual/palatal portions was removed, creating a de‐epithelialised zone that was tunnelled into buccal and palatal/lingual flaps, facilitating the adaptation and incorporation into the recipient site. This modification promoted increased contact with the underlying connective tissue, thus enhancing nutrient diffusion and graft survival. Both the palate and the MFGG were sutured using the same 5–0 nylon sutures.
– *MFGG + Xenograft (MFGG + XG; n = 22)*: Atraumatic extraction was followed by socket filling with particulate xenogeneic bone up to the interproximal bone crest limits (Bonefill Mix, Bionnovation Biomedical [0.10–1.50 mm]). The socket was then sealed using the MFGG, as previously described (Figure 1M–S).
– *Titanium membrane + Xenograft (TM + XG; n = 22) group*: Atraumatic extraction was followed by socket filling (as described above) and sealing using an anodised non‐absorbable titanium membrane (Surgitime Titanium Seal, Bionnovation Biomedical, 34 × 25 mm, thickness 0.04 mm) after buccal and lingual/palatal detachment (approximately 5 mm) to accommodate the membrane. The membrane was left exposed to the oral environment. Membrane stabilisation and suturing was performed using 5–0 nylon sutures (Figure 1T–Z).

All patients received postoperative instructions, along with analgesics (Dipyrone 500 mg, 4/4 h), systemic antibiotic therapy (amoxicillin 500 mg, 7 days, 8/8 h) and 0.12% chlorhexidine. One week after surgery, sutures in the control group were removed. For patients in the MFGG and MFGG + XG groups, sutures were removed after 14 days, whereas they were removed after 21 days in the TM + XG group.

### 2.5 CBCT Analysis

**FIGURE 2:**
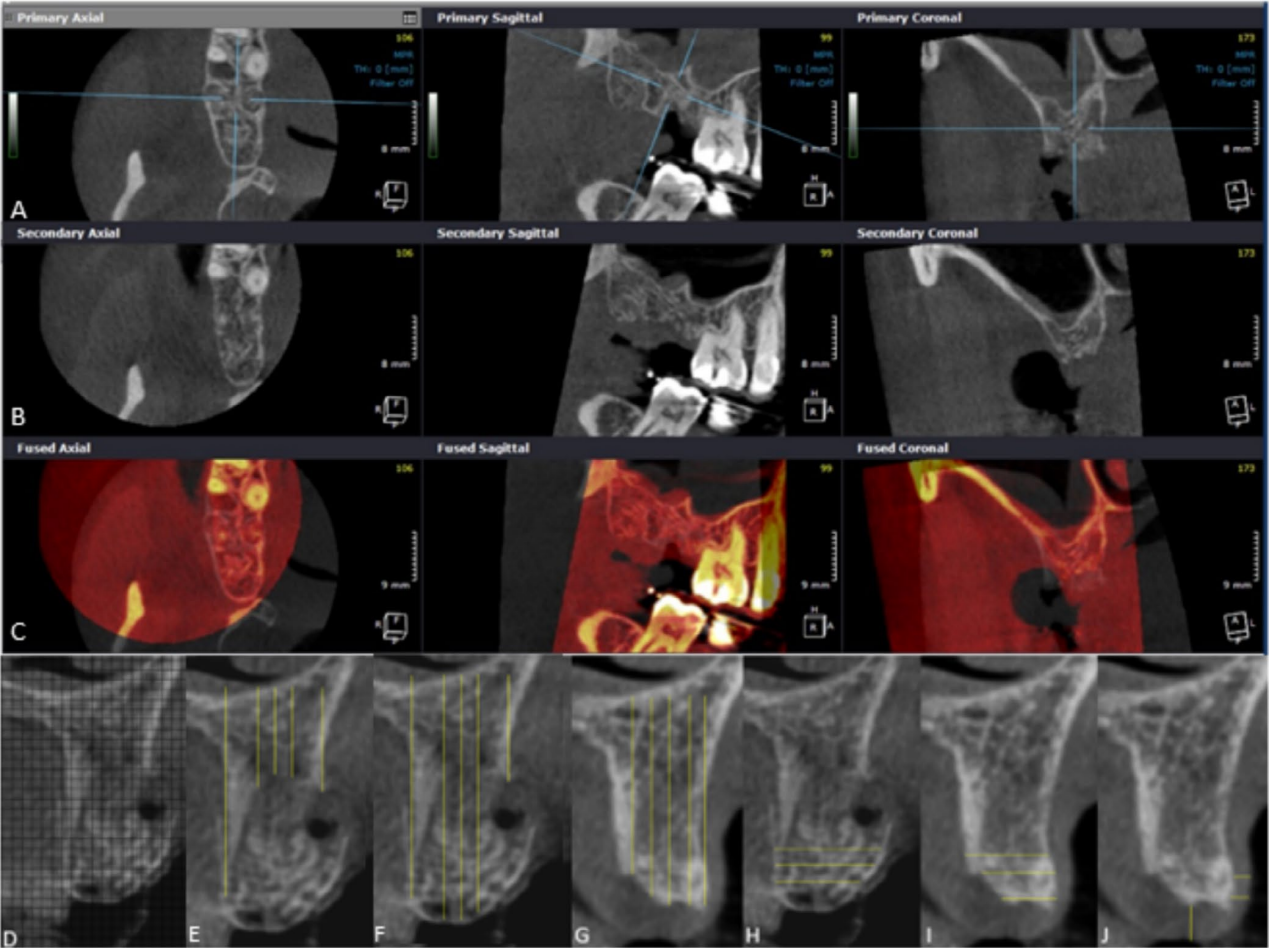
Registration of CBCT volumes of the unassisted socket healing group. The multiplanar images in the first row refer to the baseline CBCT (A). The multiplanar images in the second row refer to the postoperative CBCT (B). The images in the third row refer to the superimposition of the two volumes (C). Grid used for evaluation of the linear height and width in CBCT cross‐sections. (D) Baseline alveolar height. (E) Baseline graft height. (F) Postoperative height. (G) Baseline width. (H) Postoperative width (I). Soft‐tissue measurement (J).

A detailed description of image analysis is available in Supporting Information. Briefly, immediately after tooth extraction and also 6 months later, patients underwent cone‐beam computed tomography (CBCT) scans with lips and cheeks retracted, using the OP300Maxio device (Instrumentarium, Tuusula, Finland) with the following settings: 90 kVp, 10 mA, FOV 5 × 5 and voxel size 0.085 mm. On DICOM files, stable anatomical landmarks (i.e., the cortex of the mandibular canal) served as reference points. The ridge centre was identified on the baseline CBCT, and a cross‐sectional image through the socket centre was exported to ImageJ software, where a measurement matrix was created. Baseline and follow‐up scans were superimposed using the Fusion module in OnDemand3D software (Cybermed, Daejeon, Republic of Korea), and registration ensured that measurements were made in the same region (Figure 2A–C). Immediate postextraction linear measurements were based on socket wall limits. Using the matrix and registered CBCT volumes, the *x* (horizontal) and *y* (vertical) coordinates guided the measurements for the 6‐month analysis.

Vertical assessments were performed at the ridge centre, 2 mm from the buccal (B + 2) and lingual/palatal sides (L + 2) and at the buccal and lingual crests (Figure 2D–F). Horizontal distances were measured at the crest and at 2 and 4 mm apical to it (Figure 2G,H). Soft‐tissue thickness was also measured at the ridge centre and at 1 and 2 mm apical to the crest (Figure 2F,I). Three calibrated evaluators (M.P.L., M.T.R., T.R.V.O.) performed the vertical and horizontal measurements (inter‐examiner agreement = 90%).

### 2.6 Patient‐Centred Outcomes

Self‐reported discomfort on the first, third and seventh postoperative days was assessed using a visual analog scale (VAS). Patients were instructed to record the number of analgesics taken during the postoperative period.

### 2.7 Dental Implant Installation and Intraoral Scans

Implant planning was done using the CBCT acquired after 6 months. The patients were scanned after extraction and again after 6 months using the Primescan intraoral scanner from Dentsply Sirona (North Carolina, USA). Cone Morse implants (Biomorse XP, Bionnovation Biomedical, Brazil) were placed with a subcrestal insertion of 1 mm (M.Z.C. and R.C.V.C.). Information on the implant length, insertion torque and the need for sinus lift or regenerative bone procedures was collected.

### 2.8 Data Analysis

Initially, data were tested for normality (Shapiro–Wilk test). Demographic and clinical periodontal data were grouped and compared using the Chi‐square and Fisher's exact tests and one‐way/Tukey ANOVA for continuous variables. The repeated‐measures ANOVA/Tukey test was used to compare groups at the different timepoints regarding the vertical and horizontal dimensions. The comparison of ridge changes (Delta vertical dimensions and horizontal dimensions, i.e., 6‐month value minus baseline value) after healing, as well as patient‐centred data, was performed using the Kruskal–Wallis/Friedman tests. The occurrence of adverse events was compared using Fisher's exact test. All analyses were performed using SPSS (Statistics Software; IBM Corporation, Armonk, New York, USA), in a per‐protocol population, with a significance level set at 5%.

## 3 Results

**FIGURE 3:**
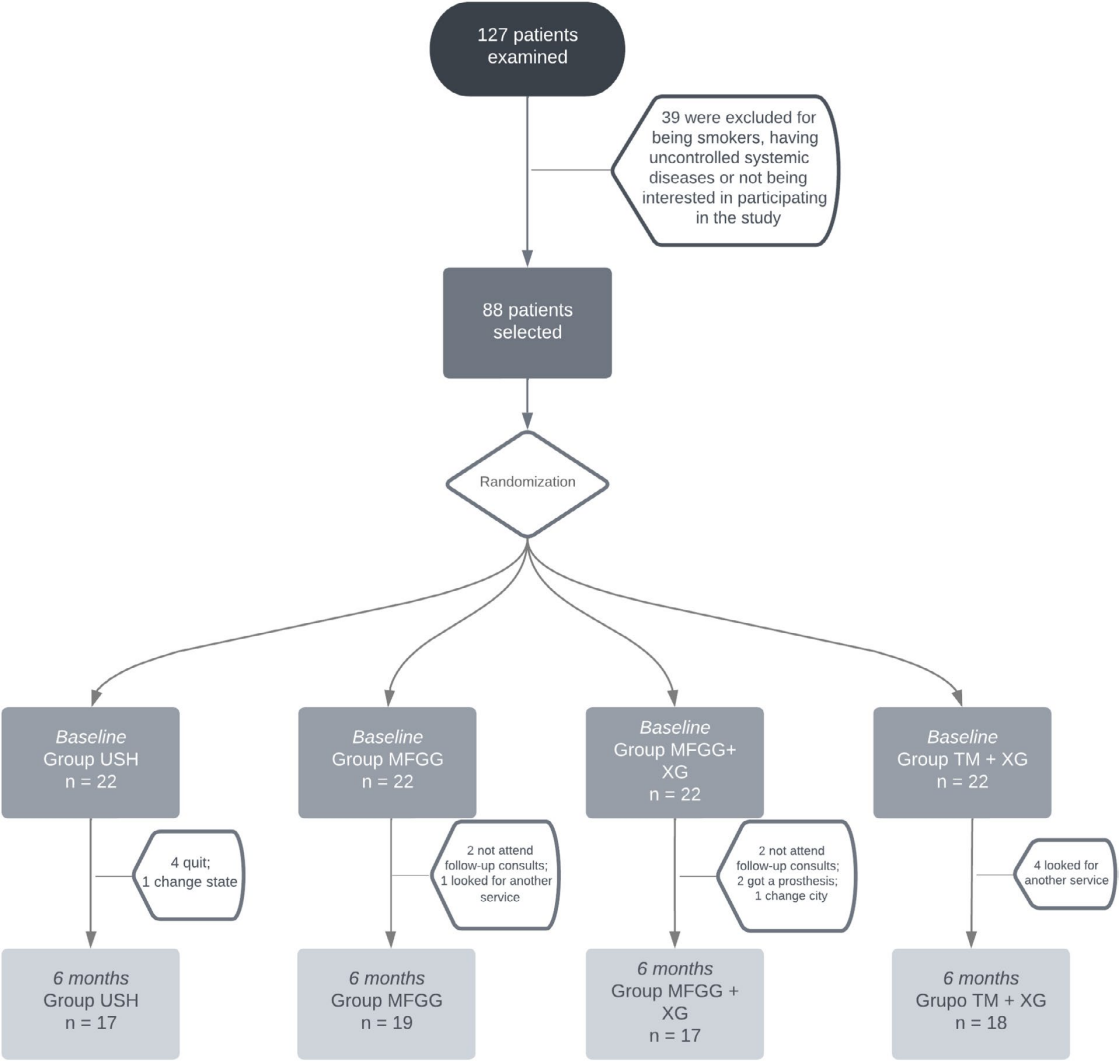
Flowchart of patients included in the study.

A total of 127 patients were examined. Thirty‐nine were excluded for ineligibility, and 17 failed to attend follow‐ups. A total of 71 patients completed the study (Figure 3); 45.1% were men, and the mean age was 48 years (22–78 years). Twenty‐three upper molars, 28 lower molars, 12 upper premolars and 8 lower premolars were included in this study. The full‐mouth periodontal probing depth among patients was 2.8 ± 0.4 mm. Reasons for extraction included fractures, cracks and extensive carious lesions. No significant differences among the groups were seen (Table 1).

**TABLE 1:** Demographic and clinical data of the population included in the study.

	Age (years ± SD)	Sex (%female)	Maxillary (%superior)	Tooth (%molar)	PI (%mean ± SD)	BOP (%mean ± SD)	PD (mm ± SD)
USH (*n* = 17)	48.4 ± 12.1	76%	53%	88%	11.0 ± 6.0	5.6 ± 2.7	2.9
MFGG (*n* = 19)	50.2 ± 11.4	56%	61%	61%	8.7 ± 5.7	4.3 ± 2.5	2.7
MFGG + XG (*n* = 17)	49.5 ± 10.0	44%	39%	61%	12.5 ± 7.9	5.4 ± 3.0	2.9
TM + XG (*n* = 18)	43.9 ± 13.0	44%	44%	83%	12.1 ± 5.4	5.6 ± 2.8	2.9
*p*	0.379	0.188	0.563	0.133	0.682	0.825	0.987

Abbreviations: BOP, bleeding on probing; PD, probing depth; PI, plaque index; SD, standard deviation.

### 3.1 Vertical and Horizontal Dimensions

**FIGURE 4:**
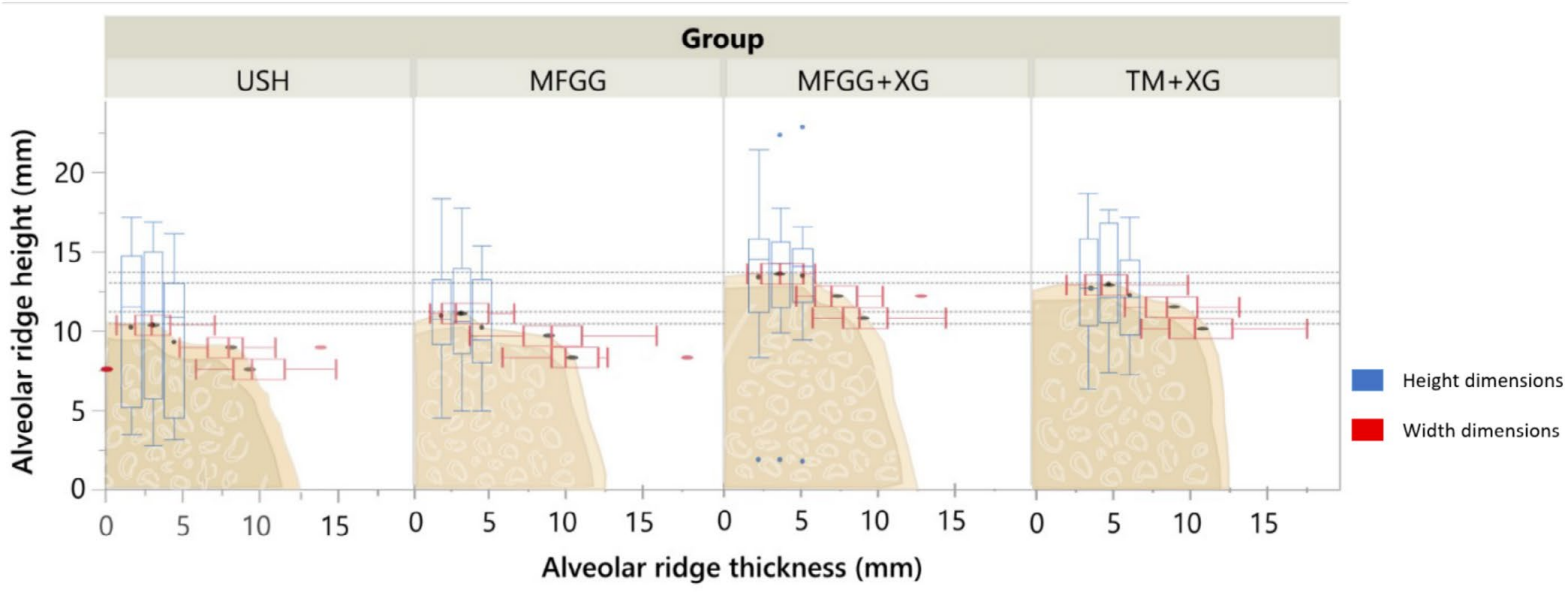
Final ridge dimensions (height and width) at the centre of alveoli for USH, MFGG, MFGG + XG and TM + XG groups.

Table 2 displays the vertical dimensions at baseline and the dimensional changes for all groups. Additionally, Figure 4 illustrates the alveolar vertical and horizontal dimensions at the centre of the alveoli, as well as the final ridge dimensions after healing in each group. There were no significant differences among the groups in baseline alveolar vertical and horizontal dimensions (*p* > 0.05). When considering the centre of the alveoli, B + 2 and L + 2 measurements, all groups showed an increase in bone height (*p* < 0.05), indicating vertical bone formation. Notably, both grafted groups (TM + XG and MFGG + XG) demonstrated more bone formation than the USH and MFGG groups (*p* < 0.05) at the centre of the alveoli and at the B + 2 and L + 2 measurements (*p* < 0.05).

**TABLE 2:** Linear dimensional changes in height and width (mm ± SD) of alveoli in all groups.

		Height linear measurement			Width linear measurement		
		B + 2	Centre	L + 2	Crest	Crest − 2	Crest − 4
USH (*n* = 17)	Baseline	5.8 ± 4.2	6.0 ± 3.8	7.0 ± 4.8	6.5 ± 4.6	10.0 ± 2.7	10.4 ± 3.3
	0–6 month change	3.3 ± 3.7^a^	4.2 ± 3.8^a^	3.1 ± 2.0^a^	−3.4 ± 3.4^a^	−2.1 ± 1.5^a^	−1.4 ± 1.2
MFGG (*n* = 19)	Baseline	5.4 ± 4.5	4.6 ± 2.7	7.4 ± 3.8	5.7 ± 6.0	10.8 ± 3.4	11.2 ± 3.2
	0–6 month change	4.7 ± 3.8^a^	6.4 ± 4.4^a^	3.4 ± 3.8^a^	−2.9 ± 5.5^a^	−2.3 ± 2.2^a^	−1.2 ± 1.7
MFGG + XG (*n* = 17)	Baseline	6.0 ± 3.8	5.2 ± 3.1	7.3 ± 4.2	5.7 ± 4.8	9.0 ± 3.8	9.5 ± 3.1
	0–6 month change	7.3 ± 3.7^a^, ^b^	8.3 ± 3.6^a^, ^b^	5.9 ± 4.4^a^, ^b^	−1.0 ± 4.8	−1.1 ± 2.8	−0.1 ± 3.5
TM + XG (*n* = 18)	Baseline	6.5 ± 3.3	5.3 ± 3.2	5.7 ± 3.2	3.9 ± 4.9	7.4 ± 5.6	11.4 ± 3.4
	0–6 month change	5.6 ± 2.2^a^, ^b^	7.5 ± 2.5^a^, ^b^	6.9 ± 2.8^a^, ^b^	0.9 ± 4.3^c^	1.6 ± 5.2^b^	−0.6 ± 1.6

*Note*: Results are expressed as mean ± standard deviation.

^a^ Statistically significant change to baseline.

^b^ Statistically significant difference to USH and MFGG groups.

^c^ Statistically significant change to USH group (*p* < 0.05).

Regarding horizontal dimensions at the crestal line, a statistically significant reduction (*p* < 0.05) was observed postoperatively in the groups without bone grafting (USH and MFGG), while the TM + XG and MFGG + XG groups maintained the initial dimension (Table 2). At Crest‐2, both grafted groups preserved the horizontal dimensions, and only the TM + XG group showed statistically superior results compared to the USH group (*p* < 0.05).

Figure 4 shows that there was a greater vertical dimension volume at the centre of the alveoli in the MFGG + XG and TM + XG groups compared to the USH group (*p* < 0.05) at 6 months, while the MFGG group did not differ significantly from either the grafted groups or the USH group (*p* > 0.05). Superimpositions of the STLs also support our findings, showing maintenance of the alveolar dimensions (Figure S3).

### 3.2 Bone Graft, Bone Crest and Soft‐Tissues Alterations

Table S1 displays the height in grafted groups at baseline and at 6 months. A significant reduction was observed after bone healing in both groups, indicating graft resorption. The TM + XG group showed the lowest percentage of crest reduction (6.0% ± 0.1%), which was statistically different from the USH group (36.6% ± 0.2%, *p* < 0.05). There was no statistically significant difference between the MFGG groups (MFGG = 27.0% ± 0.2%; MFGG + XG = 28.9% ± 0.2%) and the USH or TM + XG group (*p* > 0.05) (Figure S1). Figure S2A–C displays the occlusal and buccal gingival thickness at the top of the alveolar crest (Figure S2a) and at 1 and 2 mm below the crest on the buccal side (Figure S2B,C). No significant differences were observed among the groups, regardless of the region (*p* > 0.05).

### 3.3 Patient‐Centred and Implant‐Related Outcomes

No severe complications were reported. One patient experienced early membrane loss, and another had tissue fenestration after 16 days (11% of cases). One patient in the MFGG + XG group (6%) and three in the MFGG group (17%) lost the gingival graft during the first postoperative week. At the time of implant placement, 67% of the cases involving upper region implants in the control group required sinus floor elevation, which was statistically higher than in the TM + XG group (13%; *p* = 0.048) (Table 3).

**TABLE 3:** Patient‐ and implant‐related outcomes: Postoperative complications, sinus lifting (%), length of the implants (%) and postoperative pain after 1, 3 and 7 days in all groups.

	USH	MFGG	MFGG + XG	TM + XG	*p*
Complications (%)	0%	17%	6%	11%	0.843
Sinus lifting (%positive [*n*])	67% (6/9)A	36% (4/11)AB	29% (2/7)AB	13% (1/8)B	0.048
Implant length (%≤ 8.5 mm [*n*])	71% (12)	67% (12)	44% (8)	44% (8)	0.235
Torque (*N* ± SD)	36 ± 17	34 ± 20	47 ± 28	37 ± 17	0.338
VAS Day 1 (med [95% CI])	0B	15A	5A	5AB	0.0049
VAS Day 3 (med [95% CI])	5B	15A	15A	5AB	0.041
VAS Day 7 (med [95% CI])	0	15	5	5	0.123

*Note*: Different capital letters on the same line indicate a difference between the groups (ANOVA/Tukey and Kruskall–Wallis; *p* < 0.05).

Regarding implant length, the MFGG and USH groups had a higher frequency of implants < 8.5 mm (71% and 67%, respectively), while in the groups with bone grafting this frequency was 44% (*p* = 0.235). Patients in the MFGG and MFGG + XG groups (15 [IQR: 10–25] and 15 [IQR: 0–15], respectively) reported greater postoperative pain and discomfort on the VAS scale during the first and third days compared to the USH group (*p* < 0.05) (Table 3).

## 4 Discussion

Tooth loss disrupts periodontal tissue balance and leads to dimensional changes in the alveolar ridge (Hämmerle et al. 2011; Araújo and Lindhe 2005). ARP helps in maintaining the ridge structure and reducing the need for further procedures (Bianchini et al. 2009; Jung et al. 2004). This study assessed ARP's effectiveness and implant feasibility, comparing surgical approaches with USH in posterior regions. Bone grafting significantly preserved ridge height and crest dimensions, regardless of the use of gingival grafts or titanium membranes.

Adequate bone dimensions are key for implant success, especially in posterior regions, and USH results in greater ridge loss in molar sites (Couso‐Queiruga et al. 2021). Mardas et al. (2023) reported that molar extractions caused 1.46 and 1.20 mm reductions at the buccal and lingual sites, respectively. In our study, USH showed 2.5 mm horizontal loss and vertical reduction, reinforcing grafting benefits. Tomographic analysis confirmed that bone‐grafted groups preserved vertical dimensions better than non‐grafted groups. Xenogeneic grafts support osteoconduction and mesenchymal differentiation into osteoblasts (Lim et al. 2019; Mardas et al. 2023), preventing 1.5–2.4 mm of horizontal and 1–2.5 mm of vertical resorption (Hämmerle et al. 2011; Mardas et al. 2023).

In this study, bone grafts resulted in increased vertical dimensions, indicating new bone formation and less crestal resorption compared to USH (Avila‐Ortiz et al. 2020; Mardas et al. 2023), reaching levels close to the original crest. The vertical and horizontal dimensions, especially of the buccal crest, are key in defining the final ridge architecture (Araújo et al. 2023). Notably, graft material placed above the crest resorbed during healing. Darby et al. (2008) observed that vertical bone gain above the crest is unpredictable, although crest height tends to be maintained. Therefore, alveolar filling should stay within the crest boundaries to support the graft and blood clot. Membrane‐based socket sealing seems to improve healing by minimising buccal crest resorption (Elani et al. 2018; Lizio et al. 2014).

Intentionally exposed membranes offer a reliable strategy for dimensional preservation in posterior extractions. Expanded polytetrafluoroethylene (e‐PTFE) membranes, initially employed for submerged use, showed bacterial contamination when exposed. The development of high‐density PTFE (d‐PTFE) membranes reduced infection risks (Flemming and Scharf 2016), although they still faced bacterial colonisation during healing (Braz et al. 2024). Studies show that exposed membranes can reduce vertical bone loss by 1.20–1.83 mm compared to unassisted healing (Lizio et al. 2014; Avila‐Ortiz et al. 2020). In this study, anodised titanium membranes were used for socket sealing, effectively preventing soft‐tissue invagination. Titanium membranes help minimise resorption, especially in advanced bone loss (2.26–2.67 mm), and are a viable option for ridge preservation (Maeda et al. 2020). In addition, the TM + XG group effectively minimised buccal–lingual resorption, particularly at the crestal level. A systematic review reported that tooth extraction resulted in an average reduction of 3.8 mm in horizontal ridge dimensions (Araújo et al. 2015). In this study, horizontal measurements were standardised to precisely map changes in the coronal alveolar ridge, ensuring that the width reductions were solely due to post‐extraction resorption. The titanium membrane, even when exposed, showed greater stability of the bone graft horizontally compared to the free gingival graft, leading to more predictable ridge preservation.

Meanwhile, the use of MFGG showed favourable results, especially when paired with bone grafts. Used alone for socket sealing, MFGG offered modest improvements in bone dimensions, with no significant differences from grafted groups. However, combined with bovine bone grafts, it produced outcomes comparable to that of titanium membranes. A previous study using free gingival graft reported an additional 0.54 mm of buccal crest preservation (Karaca et al. 2015). Arroteia et al. (2023) noted that partial graft de‐epithelialisation improves vascularisation, supporting longer retention at the site. This MFGG configuration, when paired with bone grafts, yielded tomographic results similar to those with membrane sealing (Avila‐Ortiz et al. 2020; Segnini et al. 2021). Thus, in minimally traumatic extractions with intact sockets, sealing techniques such as MFGG should be considered to stabilise the blood clot, a key element for healing (Fee 2017).

ARP techniques, particularly those incorporating bone grafts, resulted in alveolar ridges with more favourable dimensions for implant placement. Implant‐related outcomes (IROs) confirmed improved feasibility and anatomical conditions. Non‐grafted groups (USH and MFGG) exhibited a greater need for sinus floor elevation (67% and 36%, respectively), while the bone‐grafted groups required significantly fewer interventions (MFGG + XG = 29%; TM + XG = 13%). These results align with those of Avila‐Ortiz et al. (2020), who reported a reduced need for additional surgeries in grafted ARP sites compared to unassisted healing. Although shorter implants and augmentation techniques may compensate for lower alveolar dimensions, such strategies are associated with a higher risk of implant instability (Do et al. 2020) and increased surgical morbidity (Hämmerle et al. 2011).

Currently, there is a growing emphasis on patient‐centred outcomes, with morbidity and postoperative pain being central considerations. We assessed the use of MFGG in ARP and demonstrated favourable clinical outcomes, particularly when combined with bone grafts. Previous studies have reported that MFGG may contribute to additional ridge preservation benefits (Hämmerle et al. 2011; Karaca et al. 2015; MacBeth et al. 2022), although its use alone did not significantly enhance vertical or horizontal bone dimensions. Harvesting palatal grafts (MFGG and MFGG + XG) was associated with increased morbidity and postoperative discomfort compared to USH, corroborating prior findings on the pain associated with palatal donor sites (Santamaria et al. 2023). Given the higher morbidity in soft‐tissue graft groups, factors like the patient's periodontal phenotype and the operator's experience should guide the use of alternatives such as membranes. Tissue fenestration in the membrane group occurred in a patient with a thin phenotype, emphasising the need for individualised technique selection.

This study has some limitations. The findings apply specifically to posterior maxillary and mandibular regions, including molars and premolars. Molars are typically multi‐radicular, increasing the complexity of alveolar socket morphology. Buccal bone wall thickness also varies, generally being thinner in the posterior maxilla. Another key anatomical factor in this region is proximity to the maxillary sinus, which may limit bone height and affect regenerative procedures (Araújo and Lindhe 2005; Barone et al. 2013). These anatomical differences must be considered when planning ARP and interpreting outcomes, and should be assessed in future research. Studies should evaluate not only outcomes but also factors such as tooth type and arch (e.g., premolar vs. molar; upper vs. lower), which may impact healing. Also, including a graft‐only group might have provided insight into the specific effects of ARP; this can be evaluated in future trials. Even though epidemiological data indicate a higher rate of inflammatory complications in implants placed in these regions (Van der Weijden et al. 2009), likely due to limited bone and reduced keratinised mucosa, gingival phenotype was not assessed. So, future research should explore its influence on ARP outcomes, particularly in posterior regions where anatomical variability can strongly affect healing. Furthermore, the long‐term impact of ARP on peri‐implant health remains inconclusive (Buonocunto et al. 2023), although benefits in marginal and interproximal bone have been noted (Beretta et al. 2021; Couso‐Queiruga et al. 2023). Lastly, the lack of a cost–benefit analysis is a limitation, as economic factors are key for clinical decision making and warrant future investigation. Also, long‐term studies evaluating not only dimensional changes but also the stability of peri‐implant tissues and clinical outcomes following ARP in posterior sites are also needed to optimise clinical protocols.

## 5 Conclusion

Alveolar preservation techniques effectively modulate dimensional changes after tooth extraction. Bone grafting is the most crucial factor in maintaining dimensions, while the choice of gingival grafts or titanium membranes for sealing depends on the clinician's experience and preference.

## Author Contributions

Leticia Sandoli‐Arroteia was responsible for the implementation of the research, recruited and prepared the patients, conducted clinical evaluations, collected the data, performed periodontal supportive therapy, took photographs and wrote the manuscript. Renato Corrêa Viana Casarin was the coordinator and designed the study, secured funding and oversaw the implementation of the research. Renato Corrêa Viana Casarin and Marcio Zaffalon Casati performed all the surgical procedures, supervised all stages of the research and contributed to the manuscript writing. Matheus Paschoaletto Lopes, Marcela Tarosso Réa and Thalita R. Vieira e Oliveira were the evaluators responsible for the tomographic analyses and were supervised and calibrated by Matheus L. Oliveira. Lucas Queiroz conducted the interpretations and superimpositions of the STLs, as well as the data interpretation. Marcelo de Faveri and Mauro Pedrine Santamaria designed the study, assisted in data interpretation and reviewed the manuscript. All authors reviewed and approved the final version of the manuscript for submission.

## Conflicts of Interest

The authors declare no conflicts of interest.

## Supporting information


**Data S1:** jcpe70004‐sup‐0001‐Supinfo.docx.

## Data Availability

The data that support the findings of this study are available on request from the corresponding author. The data are not publicly available due to privacy or ethical restrictions.
