# Supplementary material for: Dimensional Changes After Different Alveolar Ridge Preservation Techniques for Posterior Region: A Randomised Controlled Clinical Trial
Source: J Clin Periodontol. 2025 Aug 5;52(11):1584–94. doi: 10.1111/jcpe.70004 (PMC12531358; doi:10.1111/jcpe.70004)
Supplement: Supplementary file 1 — Data S1: jcpe70004‐sup‐0001‐Supinfo.docx. [file JCPE-52-1584-s001.docx]

**SUPPLEMENTARY MATERIAL: DIMENSIONAL CHANGES AFTER DIFFERENT ALVEOLAR RIDGE PRESERVATION TECHNIQUES FOR POSTERIOR REGION: RANDOMIZED CONTROLLED CLINICAL TRIAL**

**APPENDICES**

*CBCT analysis*

Immediately after the extractions, patients underwent a CBCT scan, with their lips and cheeks retracted, using the OP300Maxio device (Instrumentarium, Tuusula, Finland), following the exposure parameters: 90 kVp, 10 mA, FOV 5×5, and voxel size of 0.085 mm. A standardized method for CBCT analysis was applied. First, stable anatomical landmarks were selected, such as the superior cortex of the mandibular canal, for consistent reference points. Then, the center of the ridge was identified in the baseline (post-extraction) CBCT scan and a cross-sectional image at the center of the socket was exported to the ImageJ software. Then, a measurement matrix was created on - it consisted of a grid with precise indications of measurement locations. After 6 months, a follow-up CBCT scan was obtained using the same device and exposure parameters to ensure uniformity between the baseline and follow-up scans. These CBCT volumes were superimposed/registered using the Fusion module of the OnDemand3D software (Cybermed, Daejeon, Republic of Korea), ensuring that measurements were consistently derived from the same anatomical region. The registration process was semi-automatic: initial manual alignment was performed, followed by automatic alignment (Figure 2a–c). As a result, we obtained a follow-up image spatially aligned with the baseline scan, allowing the same measurement matrix to be applied and enhancing the precision of identifying the ridge center and ensuring consistency across all measurements.

For all groups (with and without bone grafts), immediate post-extraction linear measurements were based on the limits of the socket walls. Using the created matrix and the superimposed CBCT images, specific points were defined with x (horizontal) and y (vertical) coordinates, which served as guides to ensure that subsequent horizontal and vertical measurements were performed at the exact same locations as the initial measurements. Three evaluators (MPL, MTR, TRVO) conducted the vertical and horizontal measurements after calibration, ensuring inter-examiner consistency (ICC = 90%). For horizontal measurements, only the y-value corresponding to the greatest vertical dimension on the 6-month CBCT was used, ensuring consistent ridge positioning. Vertical measurements included the ridge center (Center), as well as 2 mm from the buccal (B+2) and lingual/palatal sides (L+2), in addition to the buccal and lingual crests (Figure 2 D–F). Horizontal measurements assessed the buccal-palatal/lingual distance at the upper ridge limits, as well as 2 mm and 4 mm apical to this level (Figure 2 G,H). At 6 months, soft tissue thickness was also measured vertically at the ridge center and 1 mm and 2 mm apical to the ridge crest (Figure 2 F, I). All steps were tested, calibrated, and performed by three trained evaluators, ensuring the reliability of the tomographic analysis.


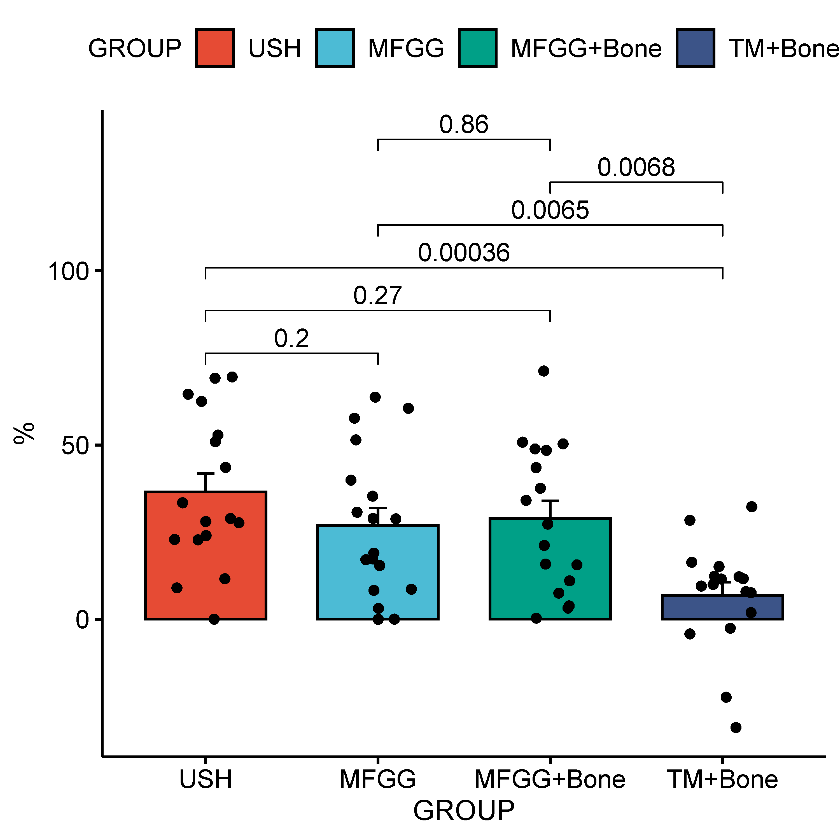


**FIGURE S1.** Percentage of resorption values in bone crest dimensions between USH, MFGG, MFGG+Bone, and TM+Bone groups. (ANOVA/Tukey, p<0.05).


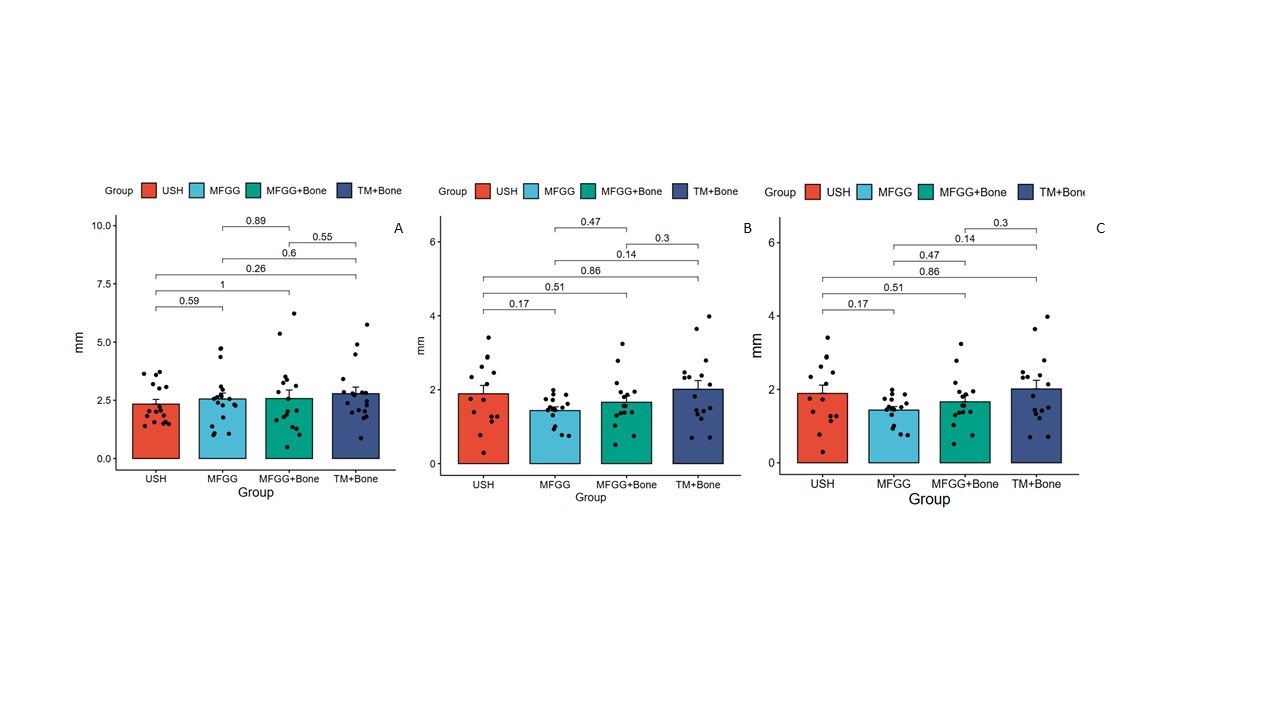


**FIGURE S2.** Soft tissue tichkness at USH, MFGG, MFGG+Bone, and TM+Bone groups after 6 months. Measurements (mm+\-sd) were taken at the center of the ridge (A) and at 1 mm (B) and 2 mm (C) apically on the buccal side (Kruskall-Wallis test, p>0.05).

**
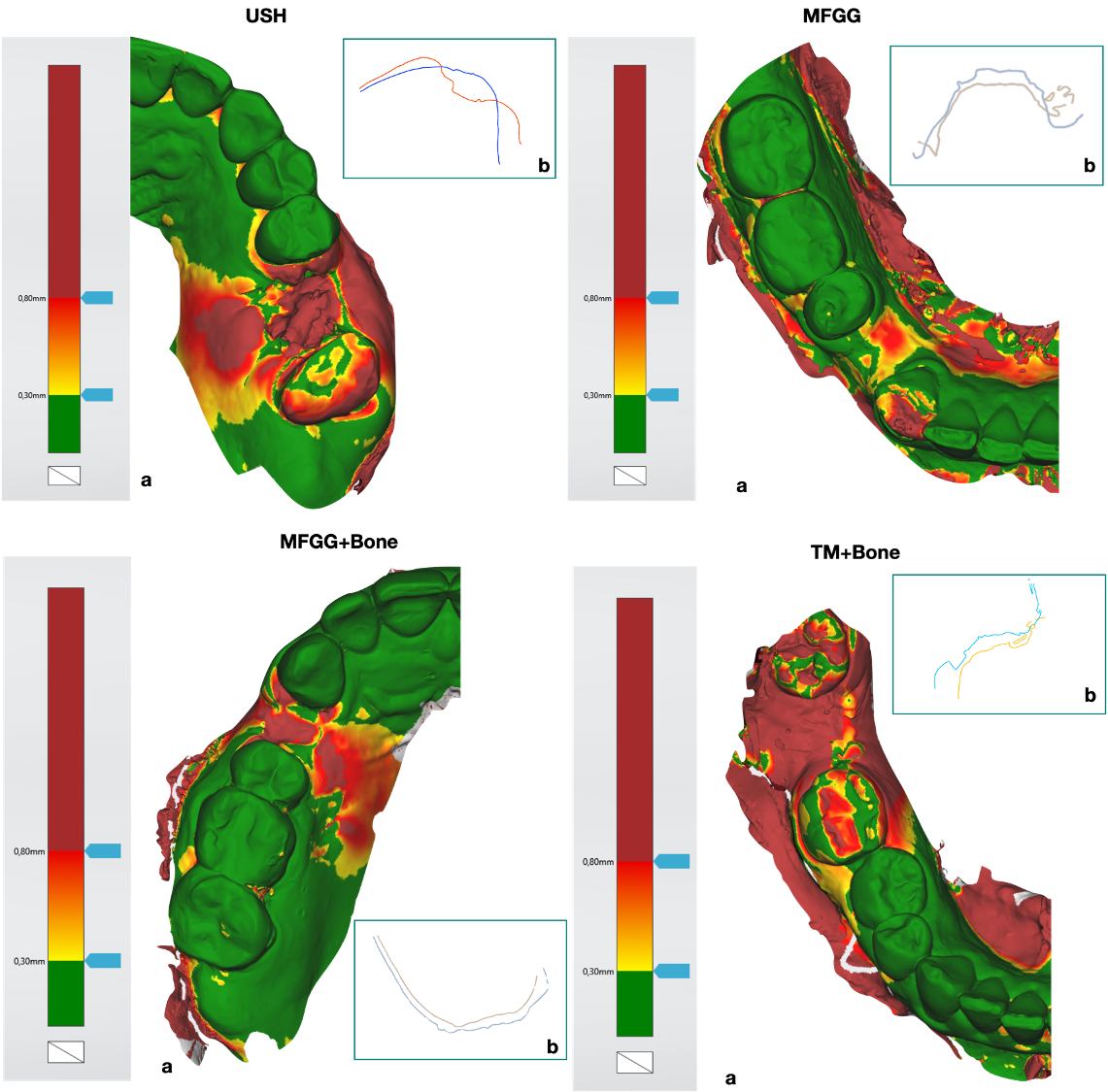
**

**FIGURE S3.** Baseline and six-month evaluations following alveolar ridge preservation (ARP) for each group: Unassisted Socket Healing (USH), Modified Free Gingival Graft (MFGG), Modified Free Gingival Graft+Bone (MFGG+Bone), and Titanium Membrane+Bone (TM+Bone). Panel (a) illustrates the superimposition of STL models with a heat map representing volumetric changes over time, while panel (b) presents contour lines delineating ridge alterations. The blue line corresponds to the baseline ridge configuration, and the yellow line represents the ridge contour at the six-month follow-up

**Table S1.** Linear height dimensions (mm±sd) in MFGG+Bone and TM+Bone, considering the bone graft volume, at baseline and 6 months in all groups and alveoli region.

|  |  | ***B+2*** | ***Center*** | ***L +2*** |
| --- | --- | --- | --- | --- |
| **MFGG + Bone**  **(n=17)** | ***Baseline*** | 14.6±3.7 | 15.0±4.9a | 14.6±4.2 |
|  | ***6 months*** | 13.3±4.2 | 13.5±4.3a | 13.2±4.3* |
|  | *0-6 month change* | *-1.4*±*1.6* | *-1.6*±*1.4* | *-1.7*±2.0 |
| **TM + Bone (n=18)** | ***Baseline*** | 14.2±3.4 | 14.7±3.9 | 14.8±4.1 |
|  | ***6 months*** | 12.1±3.0 | 12.8±3.2* | 12.6±3.5* |
|  | *0-6 month change* | *-2.1*±*1.8* | *-1.9*±*1.8* | *-2.2*±*1.9* |

*Results are expressed as mean ± standard deviation.* *statistically significant change to baseline; *^#^* statistically significant difference to USH and MFGG groups; *^$^* statistically significant change to USH group (p<0.05).
